# Identification and characterization of blocking nanobodies against human CD70: Identification and characterization of blocking nanobodies

**DOI:** 10.3724/abbs.2022141

**Published:** 2022-10-13

**Authors:** Xin Zhang, Chang Liu, Yuan Xie, Qianqian Hu, Yuanyuan Chen, Jiangwei Li

**Affiliations:** 1 Xinjiang Key Laboratory of Biological Resources and Genetic Engineering College of Life Science and Technology Xinjiang University Urumqi 830046 China; 2 Xinjiang Unique Mab BioTech Co. Ltd Urumqi 830002 China

**Keywords:** CD70, CD27, nanobody, phage display, blocking antibody

## Abstract

CD70 is overexpressed in a variety of solid and hematological tumors and plays a role in tumor proliferation and evasion of immune surveillance. Targeting and blocking its binding to the receptor CD27 have the potential to treat CD70-dependent tumors. To generate novel CD70 blocking agents, we screen a human CD70-immunized camel VHH phage display library and isolate two blocking nanobodies against human CD70 targeting different epitopes. Upon enrichment by three rounds of biopanning, two strategies are employed to identify CD70 blockers. One named affinity selection is used for detecting clones with CD70 binding by conventional PE-ELISA. However, no clone with a blocking effect is obtained from 188 enriched clones by this method. The alternative strategy named competitive selection is based on the inhibiting capacity of CD70-CD27 binding by enriched VHHs. By this method, two clones, Nb-2B3 and Nb-3B6, with strong blocking capacity are obtained from 20 enriched VHHs, suggesting the efficiency of this strategy. Furthermore, Nb-2B3 and Nb-3B6 specifically bind to CD70-positive SKOV3 and Raji cells at low concentrations. Meanwhile, Nb-2B3 has no competitive effect on the binding of Nb-3B6 to CD70, and vice versa, indicating that they target two different epitopes on CD70. Our data show that nanobodies Nb-2B3 and Nb-3B6 are potential attractive theranostic agents for CD70-expressing cancers.

## Introduction

CD70 is a member of the tumor necrosis factor (TNF) family and is transiently expressed in activated T cells, B cells and mature dendritic cells [ 1‒ 3] . CD70 mainly appears in the form of a trimer type II transmembrane protein, and its receptor is CD27. When CD27 binds with CD70, its signal transduction activates and differentiates T cells and B cells [4]. When CD27 receptors are polymerized, TNF receptor-related factors (TRAFs) and TRAF-5 adaptor proteins bind to the conserved motif of the CD27 tail and then activate the nuclear factor-κB (NF-κB) and c-Jun kinase pathways [5]. Due to the immune-stimulating function of the CD27-CD70 signaling axis, enhancing CD27 signaling has been identified as a potential treatment to promote T-cell activation and improve antitumor efficacy.

CD70 is not expressed in normal tissues and hematopoietic cells except in activated immune cells but is overexpressed in many different types of lymphoma and solid tumors [ 6, 7] . Previous studies have shown that the expression of CD70 in B-cell malignant tumors may play a role in immune escape and enhance tumor survival and growth [ 8, 9] . In non-Hodgkin’s lymphoma (NHL), CD70 ^+^ B cells induce Foxp3 expression in tumor-infiltrating CD4 ^+^ CD25 ^–^ T cells with immunosuppressive activity [10]. Blocking CD70 can significantly inhibit the NHL-mediated upregulation of Foxp3, indicating that CD70 is involved in the induction of immunosuppression. Therefore, blocking the expression of CD70 in tumor cells has become another strategy of antitumor-targeted therapy.

At present, no CD70 antibody-drug has been approved, and only a few anti-CD70 antibodies have entered clinical trials, including SNG-70 [ 11, 12] and ARGX-110 (cusatuzumab) [13]. SNG-70 is an IgG1 isotype humanized antibody which can suppress tumor growth and prolong the survival of mice with xenografted disseminated lymphoma and multiple myeloma [ 11, 12] . ARGX-110 is a llamas conventional Fab ligated with human IgG1 Fc and derived from a llamas Fab phage display library. It inhibits tumors through multiple mechanisms, including Fc-mediated cytotoxicity with enhanced ADCC and inhibition of CD70/CD27 signaling, resulting in the killing of malignant cells such as leukemia blasts and stem cells [ 13, 14] . Initial clinical trials showed that cusatuzumab had good efficacy in acute myeloid leukemia (AML) patients with low methylation drug resistance [14]. In addition, CAR-T immunotherapy targeting CD70 also has potential therapeutic effects on leukemia and B-cell lymphomas [ 15, 16] .

Although monoclonal antibodies have better performance and are successful in target-based therapeutics, they have some significant limitations such as large size, which leads to low permeability to solid tumors, complex manufacturing process, high cost of production and relatively low stability, and the only administration route is intravenous injection [17]. These limitations have created a demand to develop the next generation therapeutics which ideally combine the advantages of small molecules with the benefits of monoclonal antibodies (mAbs). Nanobodies (Nbs) naturally contain the smallest antibody fragments derived from camel heavy chain antibody (HCAb) with only a single antigen-binding domain. Besides the small size, Nbs have several advantages over conventional antibodies, including a preference to bind with epitopes not accessible to conventional antibodies, which makes them ideally suitable to generate blocking or agonist molecules, and other properties, such as high stability, ready to be engineered, and easy to be manufactured in microorganisms [18]. These characteristics of nanobodies make them well suited for the development of next generation therapeutics.

In the present study, we reported the isolation and identification of two CD70-specific nanobodies with high affinity from a human CD70-immunized camel VHH phage display library and demonstrated its strong CD70-blocking activity. At present, there is no report about CD70 nanobodies, and the nanobodies identified in our study have the potential as attractive theranostic agents for CD70-expressing cancers.

## Materials and Methods

### Bacteria, plasmids, cell lines and antibodies

*Escherichia coli* TG1 and M13K07 help phage were purchased from New England BioLabs (Beverly, USA). The pMECS phagemid was kindly provided by Professor Serge Muyldemans of the Department of Applied Biological Sciences, Vrije Universiteit Brussel (Brussels, Belgium). pET-22b and BL21 (DE3) were preserved in our laboratory. The human cancer cell lines SKOV3 and Raji were purchased from National Cell Resource in Peking Union Medical College (Beijing, China) and cultured in RPMI-1640 supplemented with 10% (v/v) fetal bovine serum (Invitrogen, Carlsbad, USA) and 1% penicillin-streptomycin (Invitrogen). CHO cells expressing human IgG-Fc fused human CD70 recombinant proteins (CD70-hFc; Cat. No. 10780-H15H3), HRP-conjugated goat anti-mouse IgG (Cat. No. SSA007) and HRP-conjugated mouse anti-HA antibody (Cat. No. 100028-MM10-H) were purchased from Beijing Sino Biological (Beijing, China). Mouse anti-human CD70 mAb (Cat. No. 355101) was purchased from Biolegend (San Diego, USA). FITC-conjugated mouse anti-HA antibody (Cat. No. A01621) was purchased from GenScript Biotech (Hong Kong, China). Cusatuzumab (Cat. No. CSD00533) was purchased from Wuhan Chemical Biotechnology (Wuhan, China). FITC-conjugated goat anti-human IgG antibody (Cat. No. F9512) and goat anti-mouse IgG antibody (Cat. No. F9137) were purchased from Sigma-Aldrich (St Louis, USA). Human IgG1 (Cat. No. GTX16193) and mouse IgG (Cat. No. GTX35009) were purchased from GeneTex (San Antonio, USA).

### VHH library construction and biopanning against CD70

Human CD70-hFc was used as an immunogen to immunize a female Xinjiang Bactrian camel subcutaneously once every two weeks for six times. All animal experiments were approved by the Xinjiang University Animal Ethics Committee. Peripheral blood lymphocyte (PBL) isolation, RNA extraction, and cDNA synthesis were performed as described previously [19]. VHH gene fragments were cloned by nested PCR with VHH back and PMCF primers described elsewhere [20]. The VHH fragments were ligated into the pMECS phagemid vector by the *Pst*I and *Not*I restriction sites. Then, the recombinant vectors were transformed into electrocompetent *E*. *coli* TG1 competent cells, and a VHH gene library was generated.

For biopanning, the phages displaying VHH genes resumed autonomous replication after infection with M13K07 helper phages at a ratio of 1:20 at 37°C for 30 min. The isolation and titration of rescued phages were performed as described previously [20]. Specific VHHs against human CD70 were enriched by three consecutive rounds of panning on 96-well microtiter plates coated with 100 μL/well human CD70-hFc at descending concentrations (50, 25, and 12.5 μg/mL). To subtract phage binding to hFc, rescued M13 phages were pre-incubated with an irrelevant protein-containing hFc before binding to CD70-hFc. After several washes with PBS and PBST, the antigen-specific phage particles were eluted with 100 mM triethylamine solution (pH 11.0) and immediately neutralized with 1 M Tris-HCl (pH 7.4). The eluted phage (output phage) titers were detected and used to infect exponentially growing *E*. *coli* TG1 strains for the next round of panning and to evaluate the enrichment of CD70-specific phages.

### Periplasmic ELISA (PE-ELISA) for selecting CD70 binders

The individual *E*. *coli* TG1-VHH clones obtained after the second and third rounds of panning were randomly picked and inoculated into wells of a 96-well round-bottom culture plate filled with 500 μL of 2YT/ampicillin (AMP)+glucose medium and cultured at 37°C overnight with shaking. To prepare nanobody periplasmic extract, 20 μL of the above overnight-grown *E*. *coli* TG1-VHH clones were inoculated into a 96-deep well plate containing 300 μL of SB-AMP medium and incubated at 37°C with shaking until OD _600 nm_ reached 0.4−0.6 (exponential phase). The expressions of VHH genes were induced by addition of isopropyl-β-D-thiogalactoside (IPTG) at a final concentration of 1 mM and overnight incubation at 30°C and 150 rpm. After cells were collected, the periplasmic proteins (nanobodies) were extracted by osmotic shock. To perform PE-ELISA for selecting the CD70 binders, 100 ng/well human CD70-hFc was coated in 96-well microtiter plates overnight at 4°C. Wells were blocked with 5% skimmed milk in PBS and incubated with the periplasmic proteins. The bound VHHs were detected using an anti-HA tag antibody conjugated with HRP. Peroxidase activities were detected by adding TMB (3,3′,5,5′-tetramethylbenzidine) substrate, and the OD _450 nm_ value was measured on a Multiskan FC microplate reader (Thermo Fisher, Waltham, USA). The 3-fold binding of VHHs with human CD70-hFc vs irrelevant proteins was defined as positive and subjected to DNA sequencing. The sequences were analyzed by IgBLAST ( https://www.ncbi.nlm.nih.gov/igblast/) and ClustalX (2.0).

### Competitive ELISA for the selection of CD70 blockers

The inhibitory effects of Nbs on the binding of CD70 to CD27 were used to evaluate their blocking capacity. A 96-well plate was coated with CD27-His (2 μg/mL). After being blocked with 5% skimmed milk and washed with PBST, the membranes were incubated with CD70 (7 μg/mL) to saturate the binding of CD70 to CD27. After washing, the cells were incubated with the periplasmic proteins, and the well without Nbs was used as the CD70-CD27 binding control. The bound CD70 was detected using mouse anti-human CD70 mAb and HRP-conjugated mouse anti-HA tag antibody. Peroxidase enzyme activity was determined after the addition of TMB as a substrate and the optical density was measured at 450 nm. The positive blockers were defined as the OD _450 nm_ value in test clones lower than 50% than that in the CD70-CD27 binding control.

### Expression and purification of CD70 nanobodies

The positive *E*. *coli* TG1 clones selected by PE-ELISA and competitive ELISA were inoculated into ampicillin-resistant SB-Amp medium and cultured to logarithmic phase. The VHH genes were expressed by addition of IPTG at a final concentration of 1 mM and overnight incubation at 30°C and 150 rpm. After the cells were collected, the periplasmic proteins (nanobodies) were extracted by osmotic shock. This periplasmic extract was loaded on a Ni Sepharose 6 Fast Flow column (GE Healthcare, Milwaukee, USA). After extensive washing, the bound proteins were eluted with elution buffer (500 mM imidazole). The purity of recombinant VHHs was evaluated by SDS-PAGE (15% acrylamide) and Coomassie brilliant blue staining.

### Antigen binding specificity of nanobodies determined by ELISA

To detect binding specificity, the structural-related proteins PD-1, PD-L1, 4-1BB, 4-1BBL, and CD27 were coated on 96-well microtiter plates at 1 μg/mL. After being blocked with 5% skimmed milk, the wells were incubated with Nb-2B3 and Nb3-B6 (3 μg/mL). The bound Nbs were detected using HRP-conjugated anti-HA tag antibody (1:3000). Peroxidase enzyme activity was determined after addition of TMB as a substrate and the optical density was measured at 450 nm. To determine the species-specific binding, nanobodies Nb-2B3 and Nb3-B6 were added to a microtiter plate precoated with human, mouse, and cynomolgus monkey CD70 (1 μg/mL) overnight. The bound nanobodies were detected as described above.

### Determination of the nanobodies affinity

The affinity of nanobodies was determined by noncompetitive ELISA according to Beatty’s method [21]. Briefly, a 96-well plate was coated with 100 μL of four different concentrations (0.25, 0.5, 1, and 2 μg/mL) of CD70-hFc antigen, and then the nanobodies at increasing concentrations (0.001−10 μg/mL) were added. After incubation, the wells were washed with PBST and incubated with HRP-conjugated anti-HA antibody (1:3000). The peroxide activity of the enzyme was detected by using TMB. The OD 450 nm was measured with a microplate reader. The affinity constant ( *K*_a_) and dissociation constant ( *K*_d_) were obtained according to the formula *K*_a_=( *n*‒1)/2( *n*Ab′−Ab). In the formula, Ab and Ab′ represent the antibody concentration (M) corresponding to the half-maximum absorbance values when the antigen concentration was Ag and Ag′, respectively, and *n*=Ag/Ag′. Then, the *K*_a_ values at *n*=2, 4, and 8 were calculated, and the average of the six *K*_a_ values was the final result. At the same time, *K*_d_ was calculated according to *K*_d_=1/ *K*_a_.

### Evaluation of Nb-2B3 and Nb-3B6 binding epitopes by competitive ELISA

To analyze the epitope specificity of the nanobodies against CD70, a competition binding assay between Nb-2B3 and Nb-3B6 was performed. The microtiter plates was coated with human CD70-hFc (100 ng/well) and blocked with 5% skimmed milk. Then, increasing concentrations of untagged nanobodies and cusatuzumab were added. For competition assays, serial dilutions (0−10 μg/mL) of HA-tagged nanobodies as competitors were added. After incubation, the bound HA-tagged nanobodies were detected using HRP-conjugated mouse anti-HA antibody. The percent difference from the competing pairs versus the Nbs alone signal was calculated using the following formula: % difference from Nb signal= [1−(signal of competing pair−no antibody signal)/(signal of binding with Nbs alone−no antibody signal)]×100%.

### Cell binding assay

Two CD70-positive cell lines, SKOV3 and Raji, were used to detect the cell binding of nanobodies by flow cytometry (BD Bioscience, Franklin Lake, USA). Cells were grown in RPMI-1640 supplemented with 10% FBS, 1% L-glutamine, and 1% penicillin-streptomycin and cultured for 3−4 days until they were 80% confluence. For flow cytometry, 1×10 ^6^ cells were collected and washed with PBS and then incubated with mouse anti-CD70 mAb or different concentrations of Cusatuzumab, Nb-2B3, and Nb-3B6 on ice for 45 min. Cells were washed, stained with FITC-conjugated mouse anti-human IgG antibody, goat anti-mouse IgG antibody, or mouse anti-HA antibody on ice in the dark for another 30 min. After extensive wash, cells were analyzed on a, fluorescence-activated cell sorter (FACS). hIgG1 and mIgG were used as the negative binding controls.

### The inhibition of nanobodies on the binding of CD70 to CD27

The inhibitory effects of Nbs on the binding of CD70 to CD27 were used to evaluate their blocking capacity. All steps in this competitive ELISA were performed similarly to the selection of CD70 blockers from periplasmic extracted Nbs as described above except that increasing concentrations of purified competing nanobodies were used. The bound CD70 was detected using mouse anti-human CD70 mAb and HRP-conjugated goat anti-mouse IgG antibody.

## Results

### Library construction and diversity evaluation

**Figure 1:**
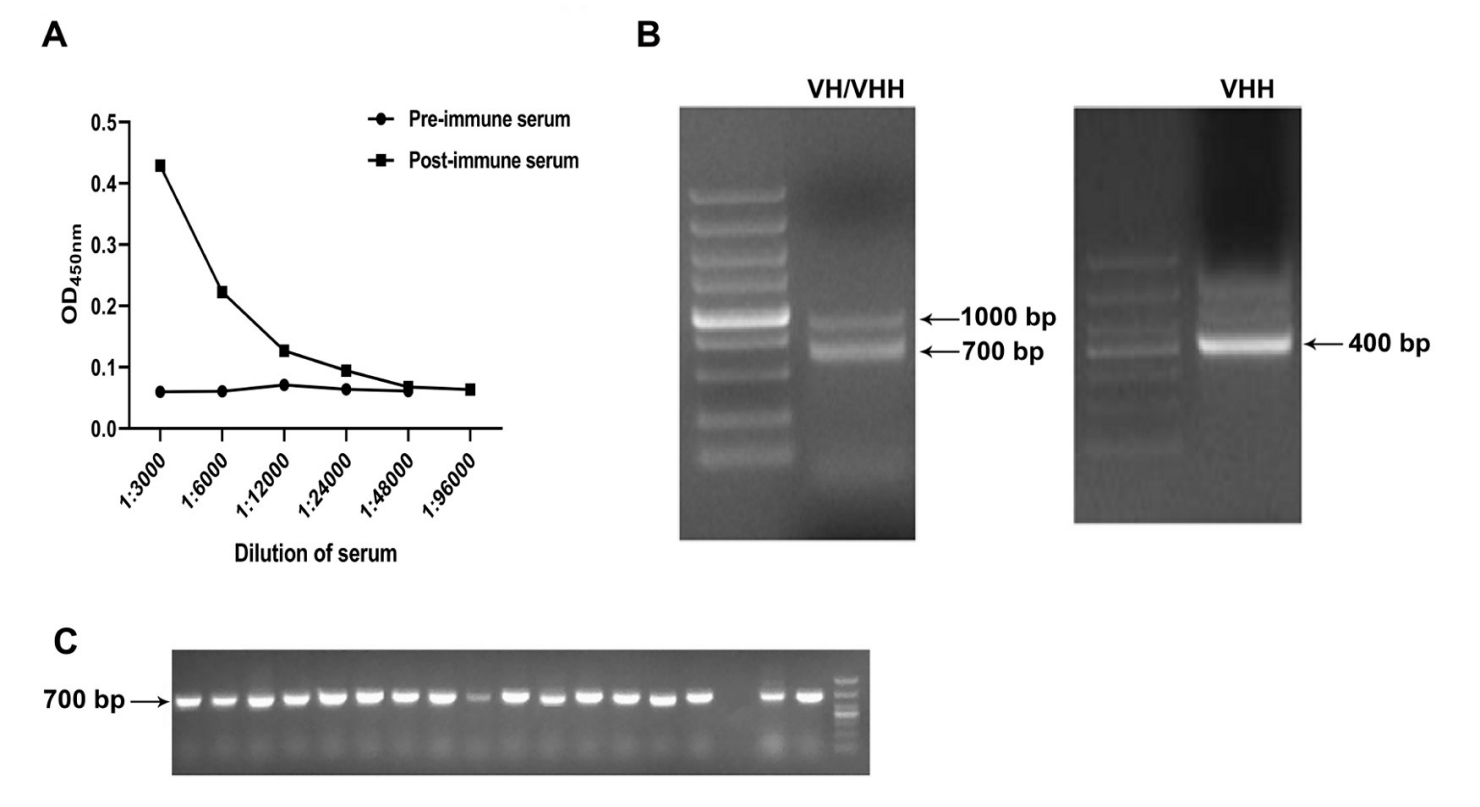
Figure 1 VHH library construction (A) The titer of CD70-specific IgG antibody in the sixth immunized serum from Bactrian camel. Data were obtained from two independent experiments. Camel serum IgG was detected using HRP-conjugated goat anti-Llama IgG (H+L) secondary antibody (immuno regents) at 1:3000. (B) VH and VHH sequences were amplified by the first (left panel) and second (right panel) rounds of PCR. The arrow indicates the VH/VHH size in the gel. (C) Detection of the VHH inserts in the library by colony PCR using 18 randomly picked clones. The positive VHH inserts have a size of 700 bp in the gel.

After six immunizations, the titer of CD70-specific IgG in camel serum reached 1:18000 ( Figure 1A). VH and VHH gene fragments amplified by first-round PCR showed sizes of approximately 1000 and 700 bp in agarose gel, respectively ( Figure 1B). The VHH gene fragments containing the FR1 to FR4 region with a size of 400 bp ( Figure 1B) was recovered by second-round PCR and ligated into the pMECS vector, transformed into *E*. *coli* TG1 competent cells, and a VHH gene library was constructed. The library has a size of 1.9×10 ^9^ colonies in serial dilution plating. The ratio of VHH inserts in the library was approximately 95%, as evaluated by colony PCR using 18 randomly selected single clones ( Figure 1C). The diversity of the library was evaluated by DNA sequencing of 34 randomly picked single clones from the library. The sequence analysis showed that 12 clones encoded the same sequence, and the remaining 22 clones represented different VHH sequences according to their CDR3 ( Supplement Figure S1), indicating medium redundancy in the library.

### Biopanning and selecting CD70 nanobodies with blocking capacities

**Figure 2:**
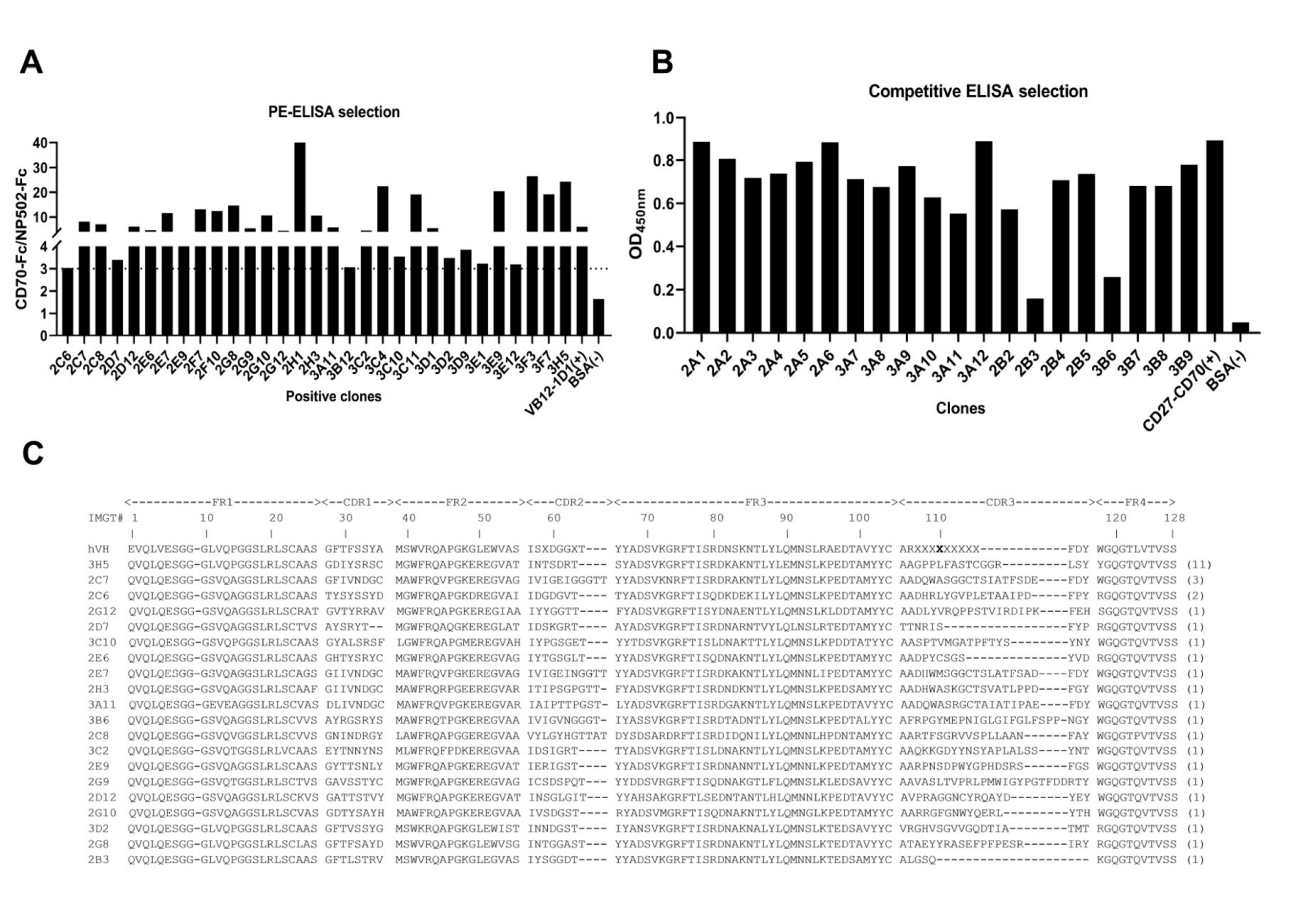
Figure 2 Selection of CD70-specific nanobodies with blocking activity (A) The detection of enriched VHHs with CD70 binding by PE-ELISA. The enriched E. coli TG1-VHH clones were induced by IPTG, and the periplasmic nanobodies were extracted and incubated with coated human CD70-hFc (100 ng/well). The bound nanobodies were detected using a mouse anti-HA tag antibody and HRP-conjugated anti-mouse IgG antibody. The binding of VB12-1D1 to its antigen was used as a positive control, and BSA was used as a negative control. (B) The ability of enriched VHHs to inhibit the binding of CD70 to CD27. The binding of CD27 to CD70 was a positive control, and BSA was a negative control. Data are shown as the mean±SD from three independent experiments ( n=3). (C) Amino acid sequences of 20 different anti-CD70 Nbs. Nbs are numbered according to IMGT numbering. The top line sequence hVH is the human germline reference sequence.

To enrich CD70-specific clones, the VHH phage display library was rescued and adjusted to a titer of 10 ^12^ pfu as the input phage. The biopanning was against coated human CD70-hFc in the well of the ELISA plate. Before each round of panning, NP502-hFc was pre-incubated with input phages to eliminate Fc binders fused to target proteins. After three rounds of biopanning, a clear enrichment occurred in round 2 and round 3 at 32- and 125-fold enrichment, respectively, compared with the last round. The titers of each input and output in three rounds of panning are shown in Table 1. The enrichment factors roughly reflect the target protein-specific enrichment. After three rounds of panning, a total of 188 clones were randomly picked and screened for CD70 binding by PE-ELISA. Thirty-two clones were identified as CD70 binding positive ( Figure 2A). Unfortunately, none of the tested clones blocked CD70-CD27 binding in the competitive ELISA ( Supplementary Figure S2). To avoid the bias of PE-ELISA in selecting CD70 blocking clones, we directly assayed the blocking activities of the enriched clones from the second and third rounds of panning by competitive ELISA using periplasmic proteins (nanobodies). We call this competitive selection. A total of 20 randomly picked clones were subjected to detection of blocking capacities in competitive selection. Two clones with over 60% blocking capacity on the binding of CD70 to CD27 were identified ( Figure 2B), suggesting the efficiency of this screening. All identified CD70 binders in PE-ELISA and competitive selection were sequenced and analyzed by IgBLAST and ClustalX (2.0) ( Supplementary Figure S3). Sequencing of these clones resulted in 20 different nanobodies, in which 3D2, 2G8, and 3B6 were derived from VH, and the rest belonged to the VHH family, as they contain the hallmark amino acid substitutions in framework 2 (V42F, G49E, L50R, and W52G) based on the IMGT numbering system ( Figure 2C). Nanobodies Nb-2B3 and Nb-3B6 showed the highest blocking activity and therefore were chosen for further analysis.

**Table:** **Table 1** Titration and enrichment of CD70-specific phages in each round of biopanning

Round of panning	Input phages ( *cfu*)	Output phages ( *cfu*)	Ratio (output/input)	Enrichment ratio
First	3×10 ^12^	4×10 ^6^	1.3×10 ^−6^	0
Second	8×10 ^12^	1.4×10 ^8^	1.8×10 ^-5^	35
Third	5.5×10 ^13^	5×10 ^8^	9.1×10 ^−6^	125

*cfu:* colony forming unit. Results are representative of 3 independent experiments.

### Expressions of CD70 nanobodies and analysis of antigen binding

**Figure 3:**
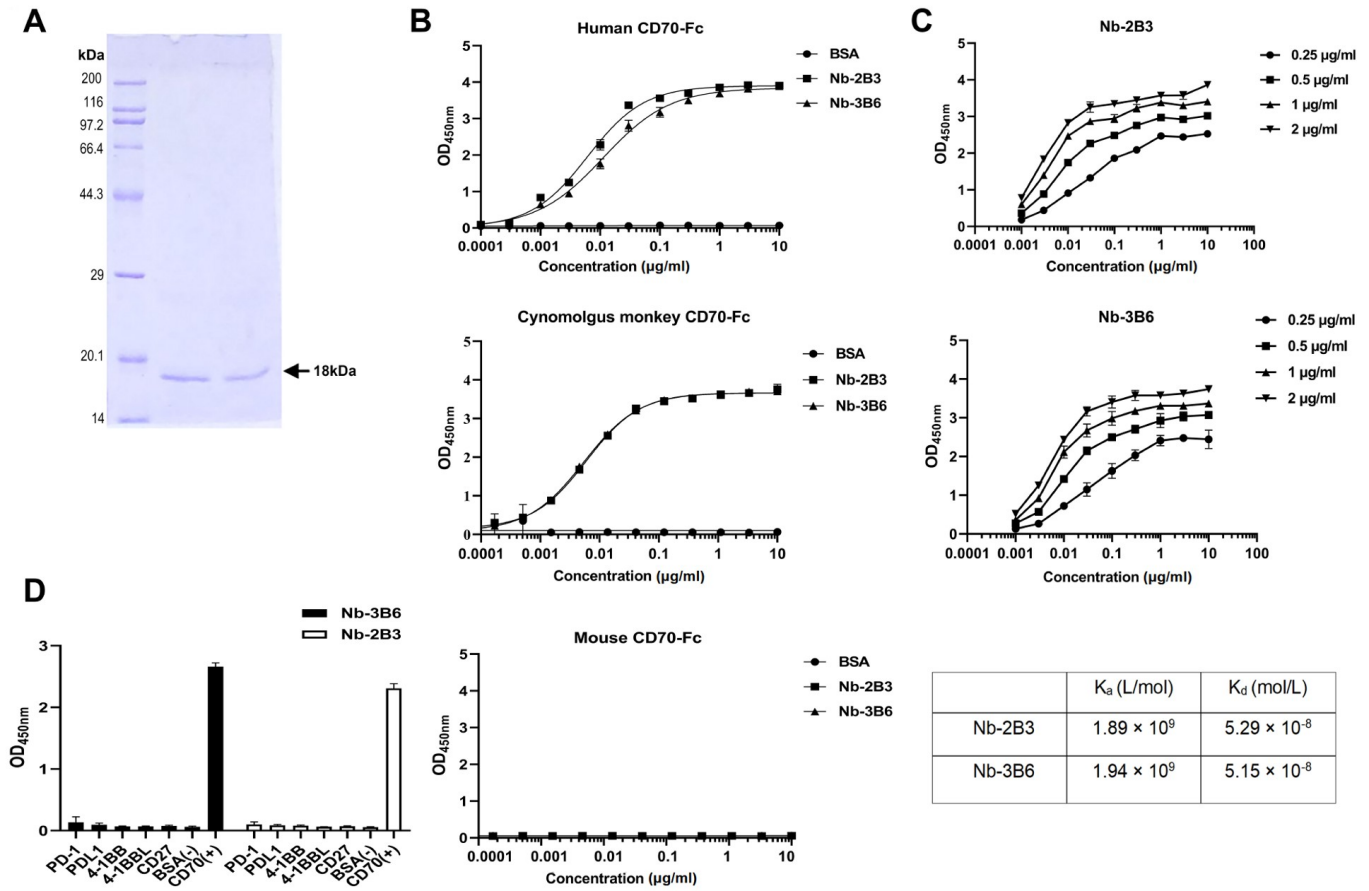
Figure 3 Protein expression and antigen-binding of nanobodies Nb-2B3 and Nb-3B6 (A) SDS-PAGE analysis of purified Nb-2B3 and Nb-3B6 proteins. (B) The binding of Nb-2B3 and Nb-3B6 to immobilized human, mouse, and cynomolgus monkey CD70-Fc. Nanobodies were incubated in plates coated with CD70-Fc from different species. The bound nanobodies were detected using anti-HA antibody conjugated with HRP. (C) The affinity of Nb-2B3 and Nb-3B6. Nanobodies at increasing concentrations (0.001‒10 μg/mL) were incubated in plates coated with human CD70-Fc at different concentrations (0.25, 0.5, 1 and 2 μg/mL). The bound nanobodies were detected using anti-HA antibody conjugated with HRP. A summary of affinity constants ( K a) and dissociation constants ( K d) is indicated in the table. (D) Binding specificity analysis of Nb-2B3 and Nb-3B6. BSA was the negative control, and CD70 was a positive control. Data in (B‒D) are OD values measured at a wavelength of 450 nm and are shown as the mean±SD from three independent experiments ( n=3).

Nb-2B3 and Nb-3B6 were chosen for expression in *E*. *coli* TG1 strains. The expressed Nbs were prepared from the periplasmic extract and purified by immobilized metal-ion affinity chromatography (IMAC). The purified Nbs reached nearly a single band at a position corresponding to approximately 18 kDa on the SDS-PAGE gel ( Figure 3A). Purified Nb-2B3 and Nb-3B6 were tested for binding to human, mouse, and cynomolgus monkey CD70 by indirect ELISA. Nb-2B3 and Nb-3B6 bind specifically with human CD70 (hCD70) and cynomolgus monkey CD70 (cyCD70) at very low concentrations but do not bind with mouse CD70 (mCD70) ( Figure 3B **)**. The EC _50_ values of Nb-2B3 and Nb-3B6 in binding with hCD70 were 0.006164 and 0.01127 μg/mL, respectively, and their EC _50_ values in binding with cyCD70 were 0.006014 and 0.005428 μg/mL, respectively. The affinity constants ( *K*_a_) were determined by noncompetitive ELISA. The nanobody binding curve under different antigen concentrations drawn according to the ELISA results is shown in Figure 3C. Nb-2B3 and Nb-3B6 bind to CD70 with a *K*_a_ (affinity constant) of 1.89×10 ^9^  L/mol ( *K*_d_=5.29×10 ^−8^ M) and 1.94×10 ^9^  L/mol ( *K _d_* =5.15×10 ^−8^ M), respectively. To evaluate the specificity, we selected some structurally related molecules, including PD-1, PD-L1, 4-1BB, 4-1BBL and CD27, to assay their binding to Nb-2B3 and Nb-3B6. The results showed that Nb-2B3 and Nb-3B6 could not bind to these antigens even at high concentrations, and they could specifically bind to CD70 ( Figure 3D).

### Nb-2B3 and Nb-3B6 specifically bind to CD70 ^+^ tumor cells

**Figure 4:**
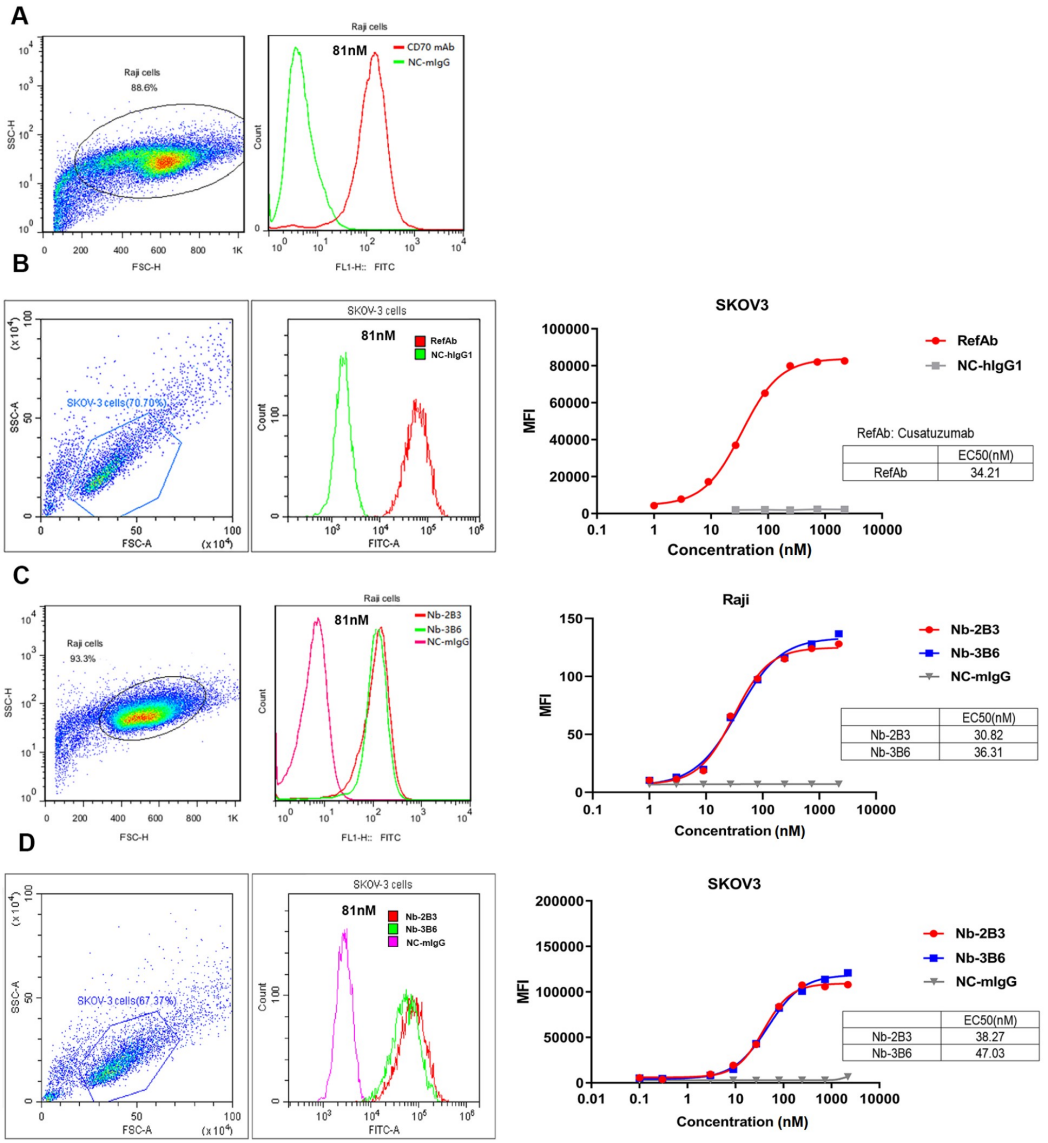
Figure 4 Binding of mAb, nanobodies Nb-2B3 and Nb-3B6 to SKOV3 and Raji cells (A) The detection of mouse anti-CD70 mAb binding to CD70 on the surface of Raji cells. Mouse IgG was used as a negative control. Mouse anti-CD70 mAb staining on Raji cells was detected using FITC-conjugated goat anti-mouse IgG antibody. (B) The detection of Cusatuzumab (RefAb) binding to CD70 on the surface of SKOV3 cells by FACS. Human IgG1 was used as a negative control. Cusatuzumab staining on SKOV3 cells was detected using FITC-conjugated goat anti-human IgG antibody. (C,D) The detection of Nb-2B3 and 3B6 binding to CD70 on the surface of Raji (C) and SKOV3 cells (D). Nbs staining on SKOV3 and Raji cells was detected using FITC-conjugated anti-HA antibody. Cusatuzumab was used as a positive control, and mIgG was used as a negative control. Data are presented as the mean fluorescent intensity (MFI) of duplicate measurements.

It was reported that CD70 is overexpressed on SKOV3 cells and Raji cells [ 22, 23] . Therefore, we chose these two cell lines to detect the cell binding of Nb-2B3 and Nb-3B6 by FACS. First, we used the widely validated humanized CD70 antibody cusatuzumab and a mouse anti-CD70 mAb to stain Raji ( Figure 4A) and SKOV3 cells ( Figure 4B), respectively. The median fluorescence intensity (MFI) of the cusatuzumab and mAb staining groups was significantly enhanced compared with that of the human IgG1 and mouse IgG staining groups ( Figure 4A,B). The EC _50_ value of the binding of cusatuzumab to SKOV3 cells was 34.21 nM ( Figure 4B). Next, the purified Nbs Nb-2B3 and Nbs-3B6 were used to test their staining on the two cells by FACS using FITC-conjugated mouse anti-HA mAb. Compared with the mIgG group, Nb-2B3 and Nbs-3B6 staining groups showed significantly enhanced MFI, and the cell-binding of Nbs was concentration-dependent ( Figure 4C,D). The EC _50_ values of Nb-2B3 and Nb-3B6 in binding to CD70 on Raji cells were 30.82 nM and 36.31 nM, respectively, and those on SKOV3 cells were 38.27 nM and 47.03 nM, respectively ( Figure 4C,D). These results suggested that the Nbs we isolated have the most similar cell-binding capacity to cusatuzumab.

### Nb-2B3 and Nb-3B6 recognize different epitopes on CD70 and strongly block CD70-CD27 binding

**Figure 5:**
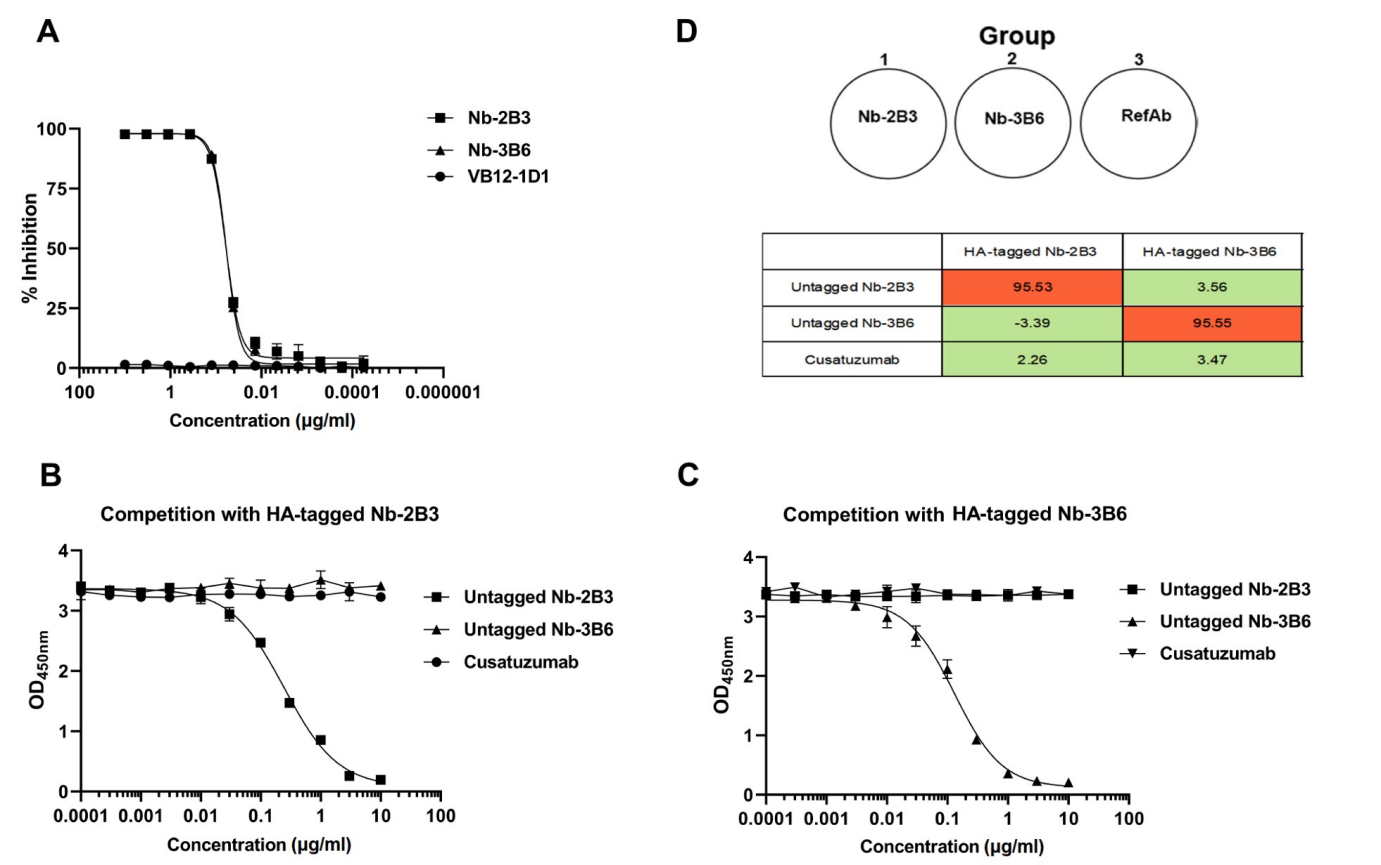
Figure 5 Competitive ELISA evaluating the blocking of CD70-CD27 binding by nanobodies (A) Blocking the activity of nanobodies. CD27 was coated on a plate, and the bound CD70 was detected using mouse anti-human CD70 mAb in the presence of different concentrations of Nbs. VB12-1D1, an irrelevant Nb, was used as a negative control. Values indicate the inhibition percentage. Data are from three independent experiments. (B,C) The binding epitopes of Nb-2B3 and Nb-3B6. CD70 was incubated with a series of concentrations of cusatuzumab, untagged Nb-2B3 (B) or Nb-3B6 (C), followed by incubation with an excessive amount of HA-tagged Nbs (3 μg/mL). Data are the OD values measured at a wavelength of 450 nm, and are shown as the mean±SD from three independent experiments ( n=3). (D) The average percent difference from the competing pairs relative to the Nbs alone signal is indicated in the table, and the three groups of antibodies (Abs) were categorized based on the binding to epitopes on CD70. The Ab-associated percentages highlighted in red are likely high-Ab competitors, and those highlighted in green are likely non-competitors.

To evaluate the blocking capacity of Nbs, we tested the binding of CD70 to CD27 in the presence of Nb-2B3 and Nb-3B6 alone and in a pairwise manner. The results showed that the two Nbs inhibited the binding of CD27 to CD70 at low concentrations and in a dose-dependent manner. The IC _50_ values of Nb-2B3 and Nb-3B6 were 0.06052 and 0.05898, respectively. At 0.3 μg/mL of Nbs, the bound CD70 was almost completely inhibited, indicating strong blocking activity of Nbs ( Figure 5A). Then, we used an ELISA-based epitope binding assay to assess the binding of Nb-2B3 and Nb-3B6 with CD70. The untagged Nb-2B3 and Nb-3B6 were used as competitors to test their inhibition of the binding of HA-tagged Nbs with CD70. Cusatuzumab was also included to examine the binding of the epitope. The results showed that the CD70 binding of HA-tagged Nb-2B3 was dose-dependent and decreased by untagged Nb-2B3 but not by untagged Nb-3B6 or cusatuzumab ( Figure 5B). Similarly, CD70 binding of HA-tagged Nb-3B6 was dose-dependently decreased by untagged Nb-3B6 but not by untagged Nb-2B3 or cusatuzumab ( Figure 5C). These results suggested that Nb-2B3, Nb-3B6, and Cusatuzumab bind to different epitopes on CD70. Based on the epitope binding results, these three antibodies can be categorized into three different groups ( Figure 5D).

## Discussion

In this study, we developed and characterized two camel-derived nanobodies that recognize different epitopes on CD70 and strongly block the binding of CD70 to CD27. To our knowledge, this is the first report about nanobodies targeting human CD70.

Therapeutic antibodies that block key immune checkpoints (such as ipilimumab and nivolumab) have opened a new era of cancer immunotherapy. Despite the successful clinical application of this therapy across different types of hematologic malignancies and solid cancers, only a small percentage of patients benefit from these therapies due to primary and secondary immune escape [ 24, 25] . Therefore, novel therapeutic target molecules involved in cancer immune escape remain to be developed.

CD70 is a recently-identified immune checkpoint molecule. The restricted expression pattern in some hematologic malignancies and solid cancers makes it the optimal target for developing antibody and CAR-T-based therapies [ 15, 16, 26‒ 28] . In addition, dysregulation of the CD70-CD27 axis within the tumor and its microenvironment is associated with tumor progression and immunosuppression [29]. It was found that overexpression of CD70 on tumors can facilitate immune evasion through CD27 expression in tumor-infiltrating Tregs. These studies highlighted the potential of CD70 blockade as an attractive strategy to suppress CD70-induced immune escape. Currently, several CD70-targeted therapeutic antibodies are under clinical trials at home and abroad ( https://www.clinicaltrials.gov/ and https://www.cde.org.cn/), including mAbs (cusatuzumab, MDX1411, and SEA-CD70), ADCs (SNG-75, SNG-CD70, AMG172, MDX-1203, and ARX305), and CAR-Ts (GIMI-IRB-20006, ALLO-316, CD70-001, and CTX130)-based drugs. The CD70-specific nanobodies (or VHHs) we isolated in this study are structurally different antibodies from traditional mAbs. Because of their unique biophysical and pharmaceutical properties, Nbs have wide application in drug development and diagnostics [ 30‒ 34] . To obtain CD70 blockers, we employed two strategies for screening and isolating Nbs from the VHH display library. As the affinity between CD27 and CD70 is unusually higher ( *K*_d_=10 ^−7^ M) than that between general receptor and ligand [35], a CD70-CD27 blocker must access the interface of CD70/CD27 and bind to CD27 more strongly than CD70. Nbs usually possess long CDR3 and preferably bind buried epitopes in clefts on protein surfaces, which are usually not accessible to conventional antibodies. Accordingly, in addition to affinity-based PE-ELISA screening, we also employed function-based competitive ELISA screening and isolated two potent CD70 blockers from a small number of candidate clones, confirming the potency of hidden epitope accessibility of Nbs.

Nb-2B3 and 3B6 are well expressed not only in the periplasm in *E*. *coli* TG1 but also in the cytoplasm in *E*. *coli* BL21 (DE3) strains (data not shown), suggesting their good solubility. Two Nbs bind with both human and cynomolgus monkey CD70 but not with mouse CD70, and there is no immunological cross-reaction with several structurally-related immune checkpoint proteins. Furthermore, Nb-2B3 and Nb-3B6 bind well with two tested CD70-positive malignancies and solid cancer cells, and blocking the CD70-CD27 axis may inhibit CD27 signaling. Importantly, Nb-2b3, Nb-3B6, and Cusatuzumab have similar cell-binding abilities but bind to three different epitopes of CD70.

One of the limitations of Nbs in therapy is their shortage of effector elements. Generally, the human IgG Fc fragments were added to the C-terminal in Nbs, resulting in ADCC, CDC, and ADCP effects, such as Cusatuzumab [13]. However, the molecular size of the Nb-Fc fusion increased 3 times compared with that of Nbs, which might lead to reduced tumor tissue penetration. Other strategies to acquire antitumor effects include conjugating chemicals and fusing immunotoxins [ 36, 37] , linking with CD16 VHH [38] and recently-reported endogenous antibody-recruiting nanobodies [39]. The other limitation of Nbs in therapy is their short half-life. Because of the small molecular size of Nbs, they are rapidly eliminated from the blood by renal filtration [40]. These limitations of Nbs should be overcome for their effective applications in therapy.

However, from another perspective, the single domain feature of Nbs makes them optimally applicable in CAR-T cells. Recently, several CD70-targeted CAR-T therapies have been reported, and a few are under clinical trials [ 25– 29] . CD70-targeting CAR-T therapy is effective against CD19-negative B-cell lymphoma and gives rise to an alternative treatment for CD19-negative cancers. Although all reported antibodies used in CAR-T therapy are scFvs, their function is limited by their potential immunogenicity, poor binding capacity, instability, and tendency to form aggregates *in vivo*. Given their excellent properties, nanobodies are a good alternative for scFv in chimeric receptors. CD70 is expressed in hematologic cancers as well as in solid tumors. Therefore, Nbs-based CD70 target CAR-T therapy would be potent in treating solid tumors. Furthermore, by binding to different epitopes, Nb-2B3 and Nb-3B6 can be developed into a bivalent nanobody-CARs module to enhance their effects.

In summary, the two Nbs we isolated target different epitopes of CD70 and possess high CD70-binding capacity and CD70-CD27 blocking potency. They have potential use as attractive theranostic agents for CD70-expressing cancers.

## Supplementary Data

Supplementary data is available at *Acta Biochimica et Biophysica Sinica* online.
